# Cycling injury risk in London: A case-control study exploring the impact of cycle volumes, motor vehicle volumes, and road characteristics including speed limits

**DOI:** 10.1016/j.aap.2018.03.003

**Published:** 2018-08

**Authors:** Rachel Aldred, Anna Goodman, John Gulliver, James Woodcock

**Affiliations:** a University of Westminster, United Kingdom; b London School of Hygiene and Tropical Medicine, United Kingdom; c Imperial College, London, United Kingdom; d Centre for Diet and Activity Research, University of Cambridge, United Kingdom

**Keywords:** Cycling, Injury, motor traffic, Risk, Safety in numbers

## Abstract

Cycling injury risk is an important topic, but few studies explore cycling risk in relation to exposure. This is largely because of a lack of exposure data, in other words how much cycling is done at different locations. This paper helps to fill this gap. It reports a case-control study of cycling injuries in London in 2013–2014, using modelled cyclist flow data alongside datasets covering some characteristics of the London route network. A multilevel binary logistic regression model is used to investigate factors associated with injury risk, comparing injury sites with control sites selected using the modelled flow data. Findings provide support for ‘safety in numbers’: for each increase of a natural logarithmic unit (2.71828) in cycling flows, an 18% decrease in injury odds was found. Conversely, increased motor traffic volume is associated with higher odds of cycling injury, with one logarithmic unit increase associated with a 31% increase in injury odds. Twenty-mile per hour compared with 30mph speed limits were associated with 21% lower injury odds. Residential streets were associated with reduced injury odds, and junctions with substantially higher injury odds. Bus lanes do not affect injury odds once other factors are controlled for. These data suggest that speed limits of 20 mph may reduce cycling injury risk, as may motor traffic reduction. Further, building cycle routes that generate new cycle trips should generate ‘safety in numbers’ benefits.

## Highlights

• This paper investigates cycling injury risk in relation to exposure, in London.
• Study benefits from spatially detailed models of cyclist and motor traffic volume.
• Residential or 20 mph streets have lower injury odds than other street types.
• Bus lanes have no impact on cycling injury odds, after controlling for other factors.
• Increasing cyclist and reducing motor traffic volumes or speed limits reduces risks.

## 1 Background

### 1.1 Factors contributing to cycling injuries

Policy-makers in many cities seek to increase cycling from a low base, yet struggle against the perception that cycling is risky. In London, cyclists have an eight-fold over-representation in casualty figures, compared to their mode share of ∼2% (TfL, 2011), and risk is substantially higher than in the Netherlands (Woodcock et al., 2014). Infrastructure, vehicle design, and road user behaviour can all contribute to injury (e.g. on cyclist behaviour, Pai and Jou, 2014, on driver behaviour, Johnson et al., 2014; on HGVs and cyclist injury, Morgan et al., 2010). Transport authorities can directly modify road infrastructure, for instance via building cycle paths. They also seek to indirectly modify vehicle design and road user behaviour, via for instance legal or regulatory changes (e.g. 20 mph speed limits to reduce traffic speeds, or mandatory sideguards on large vehicles to reduce crush injuries), enforcement or education, or ITS systems alerting drivers to the presence of vulnerable road users (Silla et al., 2017).

### 1.2 Cycling injuries and the road environment

Many existing studies focus on characteristics of the road environment that can be directly measured. The usual approach is to analyse the characteristics of sites where vulnerable road users are injured (e.g. Jerrett et al., 2016), with much work comparing characteristics of serious and slight injury sites (e.g. Kaplan et al., 2014; Chen and Shen, 2016). However, as Dozza (2017) comments, relatively little transport research analyses injury site characteristics in relation to exposure, i.e. the volume of use of each road segment that gave rise to a given number of injuries (Dozza, 2017; Vanparijs et al., 2015). For example, in London two-thirds of cyclist injuries take place on primary (‘A’) roads; but does this represent a higher *risk* associated with cycling on primary roads, or merely the presence of a larger number of cyclists? Much transport research has been unable to answer such questions, due to not controlling for exposure (Vandenbulcke et al., 2014). This has tended to happen because data on exposure has often been limited or absent.

### 1.3 Studies controlling for exposure: Individual-level

Studies incorporating exposure deal with the problem in different ways. One approach is to use individual-level data, via a case-crossover approach. Participating injured individuals are treated as their own controls, with control sites (for comparison with injury sites) selected from the routes that they were following prior to injury. While the design is relatively rigorous it involves conducting large-scale new empirical research. This is often not feasible; Teschke et al. (2012) is a relatively rare example. This Canadian study used hospitals to recruit participants, a notable finding being substantially reduced injury odds associated with cycle tracks separated from motor traffic.

### 1.4 Studies controlling for exposure using aggregate data

Other studies use aggregate data for a small number of sites, utilising existing count data or collecting bespoke new count data to analyse in relation to administrative injury data. These studies often focus on major roads and/or intersections, limiting the characteristics that can be compared. Key findings often highlight the impact of cyclist and motor vehicle volumes. Miranda-Moreno et al. (2011) found a ‘safety in numbers’ effect (more cyclists, less risk per cyclist) and that growth in motor traffic was associated with increased cycling injury risk. Nordback et al. (2014) found a non-linear relationship to cyclist injury for both motor and cycle traffic, with particularly high risks at intersections with under 200 cyclists per day.

Studies covering the whole route network can compare a greater diversity of route types. However, often there is insufficient data on cycling volumes, cycling infrastructure, and/or route environment across the network. Two recent examples are Williams (2015) and Vandenbulcke et al. (2014), in New Zealand and Belgium respectively. These use a case-control method, comparing injury sites with control sites selected based on aggregate cycling volumes across the network. With the growing quality and quantity both of GPS data (Whitfield et al., 2016) and infrastructure and road network data, this literature is likely to grow. At present literature is relatively limited and findings sometimes conflict. Williams (2015) found presence of a cycle lane reduced injury odds, while growth in motor traffic, driveways, and intersections increased them. Vandenbulcke et al. (2014) found increased risk associated with factors including on-road tram tracks, bridges without cycling infrastructure, complex intersections, shopping centres or garages, and heavy van and truck traffic.

Neither Williams (2015) nor Vandenbulcke et al. (2014) included the impact of cyclist volumes (i.e. a possible ‘safety in numbers’ effect) in their model.^1^ However, this means that any ‘safety in numbers’ impact due simply to the presence of more cyclists on a route segment (as per Miranda-Moreno et al., 2011) cannot be untangled from the impact of built environment or road conditions. Yet it would be useful to separate these in analysis. Otherwise, we might assume a type of infrastructure is intrinsically safer, yet this might be safer because (in the context under study) more cyclists use that type of infrastructure.

Summarising, relatively little literature analyses injury risk relative to cycling flows, and it is particularly unusual for studies to incorporate both infrastructural variables and the potential ‘safety in numbers’ effect. Most studies have relatively limited data on motor traffic volumes (data is often only available for main roads) which along with cycling volumes is the most frequently studied variable in relation to cycling injury risk. Results from the literature remain mixed (for instance, Teschke et al., Williams, and Vanderbulcke et al. finding differing results related to cycling infrastructure) and there is a clear need for more research that can incorporate per-user risk, road infrastructure characteristics, and the impact of cyclist and motor traffic flows. The aim of this paper is to provide such an analysis, allowing the separation of ‘safety in numbers’ effects on cyclist injury risk from the impacts of some characteristics of the road environment including speed limits.

## 2 Methods

### 2.1 Approach and data sources

The current paper makes use of one city whose transport authority has – unusually – developed a model of cycling flow across the network. This provides findings for London not previously demonstrated. However, there are wider implications. Methods here could be used in other localities who have developed or are developing a cycling model, or where sufficient quantity and quality of GPS data allows aggregation of this to provide a similar flow map (see e.g. Strauss et al., 2013). Different or additional infrastructural and road environment datasets could be used in future, for instance generated through use of Google Streetview.

This paper uses the case-control method applied by Vandenbulcke et al. (2014) and Williams (2015), a method common in epidemiological research. Sites where an injury did occur were compared to ‘control’ sites randomly selected based on cycling volumes. Control sites represent an expected outcome if injury risk was distributed randomly across all cyclists on all parts of the network, without any effect of infrastructural characteristics and road environment. Statistical modelling was then used to establish the extent to which different factors are associated with elevated or reduced injury odds in the given context. See Table 1 below for a list of variables used in the model and their source.

**Table 1:** Datasets Used.

Variables	Dataset and Source
Injury (dependent variable, 0 if control point; 1 if injury point)	Transport for London’s Cynemon cycle traffic model, base year 2014, for control points;Department for Transport road injury data (Stats19) for 2013–2014, for injury points

Independent variables	
Cycling flow (logged)	TfL’s Cynemon cycle traffic model, base year 2014
Motor traffic flow (logged)	Imperial College London (ICL) motor traffic model, base year 2014
Road Class (5 categories)	Imperial College London (ICL) motor traffic model, base year 2014
Junction Status (Yes/No)	Ordnance Survey ITN highway network
Bus Lane (Yes/No)	TfL London Bus Network 2013
Speed limit (20 mph/30mph/ 40mph+)	TfL London Speed Limit Map 2014
Index of Deprivation (deciles)	Indices of Deprivation by LSOA, via London Datastore

### 2.2 Exposure estimation

Transport for London (TfL) has recently developed a model named Cynemon, which estimates daytime weekday cycling flow across the London route network. Developers used a variety of input data to build, calibrate and validate the model (TfL, 2017). These include Census data, Strava Metro data, TfL and DfT count data, and new survey data used to develop a route choice algorithm. Approximately two thousand five hundred cycle counts across London were used in model calibration. The base year, used here, is 2014.

Cynemon estimates cycling flow across most of the London route network (i.e. not only primary roads, traditionally the focus of strategic transport models), for morning peak, interpeak, and evening peak, on weekdays, for all trip purposes.^2^ It is a strategic model with some links excluded due to low importance for cycling, while others have a modelled cycling flow of zero. Cynemon’s routing algorithm gives the greatest weight to route directness but is also partly dependent on infrastructure variables. This algorithm is based on the revealed preference study conducted for the Cynemon build, calibrated on observed count data, and supported by literature suggesting cyclists do not detour far off the shortest route (Winters et al., 2010).

Data used from Cynemon for this study consisted of modelled daily two-way cycling flow on weekdays between 7 a.m.–7 p.m., for each included link. First, any excluded and zero-rated links were removed. The remaining network covered around a third of the London route network but accounted for 89.5% of reported cycling injuries occurring weekdays 7 a.m.-7 p.m.. SPSS’s Complex Samples tool was used to sample from this dataset, with each section weighted by a volume factor derived by multiplying the length of the section by the two-way modelled cyclist flow across the day.^3^ Proportional Probability Sampling was used allowing replacement (i.e. any individual section could be associated with more than one control event, as well as with injury events). This produced a set of 6600 control sections.

Once this had been done, control sections were turned into points using QGIS^4^’s random selection tool, having buffered the Cynemon network to 12 m, which allowed the control points to fall either side of the central line representing each link (as do injury points). Mapping to Ordnance Survey ITN (Integrated Transport Network) Layer left 6046 control points remaining (91.6%); i.e., excluding control points falling on completely off-highway links such as shared-use paths along canals.

**Fig. 1:**
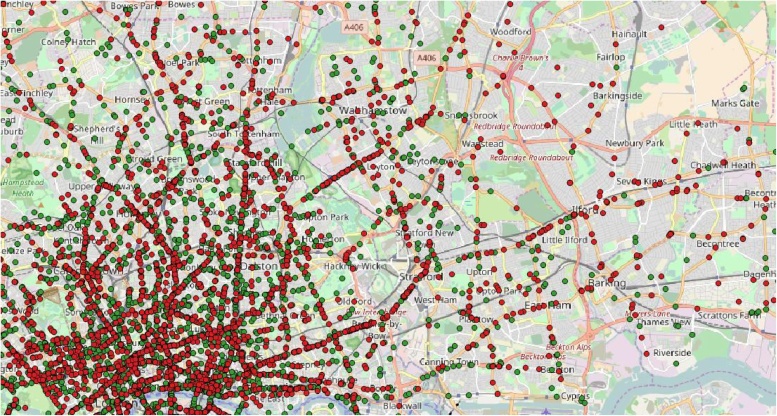
Injury (red) and control (green) points in North-East London, OpenStreetMap base (©OpenStreetMap contributors) (For interpretation of the references to colour in this figure legend, the reader is referred to the web version of this article).

Injury data comes from Stats19 police data from 2013 and 2014, used for comparability with Cynemon.^5^ Out of an initial 9769 cycle injuries, 2790 were excluded that did not take place on a weekday between 7 a.m. and 7 p.m., then a further 735 that did not match to ITN or Cynemon networks (the latter defined as links with valid flows >0). This left 6244 cycle injury points eligible for analysis, of which 16 were fatal, 545 serious and 5683 slight. Combined with the 6046 control points this yielded a dataset of 12,290 combined control and injury points then used in further analysis. Fig. 1 illustrates control (green) and injury (red) points in North-East London.

### 2.3 Risk factors

Risk factors considered are road classification, motor traffic volumes, cycling volumes, junctions, Inner or Outer London location, speed limits, local cycling prevalence and deprivation, and presence of bus lanes. Bus lane data were sourced from a TfL dataset provided for 2013; with little change in 2014.^6^ The use of cycle infrastructure data from OpenStreetMap was attempted, but proved unreliable: a random test of points on the OSM cycle-track network found no visible infrastructure present at half of such points.^7^

A model created by Morley and Gulliver (2016) was used to derive estimates of motor traffic volumes, and for road classifications. This was originally built to investigate noise pollution across the UK, with estimates for all public roads. The model was created in 2014-5 using 2013 DfT count data and additional local count data. It is referred to here as the ICL (Imperial College London) model. See Table 1 for datasets used in relation to modelled variables.

### 2.4 Identifying location characteristics

The three core datasets – ITN, Cynemon, and ICL – are not identical networks. ICL is based on OpenStreetMap crowd-sourced data, while ITN is produced by Ordnance Survey. While Cynemon was based on Ordnance Survey data, this was simplified and some road links do not map onto ITN. Hence, matching points to these three datasets – generating the key variables of junction status, road type, cycling flows, and motor vehicle flows – was done separately, based initially on closest match by distance.

Junction locations were identified as where three or more sections joined (based on overlaps with buffered points). Comparing the proportion of junctions identified from ITN to the proportion of injury incidents reported as within 20 m of a junction by police officers, proportions were similar (86% identical).^8^ Junction points were then treated differently from links when looking up characteristics from route segment datasets covering the whole network. This was because assigning closest match by distance on such networks under-represented primary roads at junction locations, due to incorrect assignment to side road rather than main road links.

For the junction points, the bias towards side roads was corrected for ITN and ICL, which have an attribute denoting road type. This was done by searching for the closest primary road section to each junction point. Where this fell within 12 m of the point in question (a figure decided upon by testing the subsequent ratios of primary roads to non-primary roads, and comparing the results to those in Stats19), it was selected in preference to the existing identified section, if this was different. This produced a road type split much closer to Stats19 than initial ‘distance only’ matching, with agreement in 93% of cases. The same approach was followed for the speed limit data which is a map of the whole of London with road type data. The Cynemon dataset used did not contain road type, nor could it easily be matched to other networks. Instead sensitivity testing was used to examine the potential impact of side road bias, by calculating mean cycle volume for all links at junction sites.

Matching points to the bus network simply involved identifying whether a point was close enough to each network (using being within 16 m as the criterion, based on manually checking categorisation of a sample) to be on it. Because this network is sparse, the ‘side roads’ issue is unproblematic. Area-based measures were straightforward, with QGIS was used to determine (i.e. spatial join) the area and hence area characteristics associated with each point.

### 2.5 Statistical analyses

Two-level random intercept logistic regression models were fitted, with points (level 1) nested within boroughs (level 2). The outcome was whether a point was an injury site, as opposed to a control site. A fixed-effect model was fitted at the points level, plus a random intercept for each borough. For the fixed-effect part of the model, a hierarchical approach to modelling was used, guided by a conceptual model sequentially adjusting for:

1 Area characteristics: region of London and small-area income deprivation
2 Road characteristics: road type, speed limit, bus lane, junction status
3 Travel volume: motor vehicle volume on road segment, cycle volume on road segment.

It was determined *a priori* to test for interactions between junction status and each other variable in the model in turn. Area deprivation was entered as a linear term due to no evidence of non-linearity, as judged by including a quadratic term (p = 0.4). Motor vehicle volume and cycling volume were entered as continuous variables after first taking the natural logarithms.

After first fitting models containing all 12,290 points, regression models were then fitted for only the 6244 injury points, with outcome being whether the injury was KSI (killed or seriously injured) or not. This was to examine whether the predictors of KSI injuries differed from those of slight injuries. The Supplementary Material also presents models comparing only KSI injuries with the control points.

All analyses used Stata 14.1.

## 3 Results

### 3.1 Descriptive characteristics of injury and control points

Table 2 presents descriptive characteristics of injury and control points, with some differences immediately apparent. For example, while around a quarter of cycling takes place on streets with under 2000 motor vehicles per day, such roads only account for around one in eight injury points – i.e. 50% of what would be expected if those roads were as risky/safe as roads with more motor traffic. Similarly, the control point distribution suggests that although nearly 30% of cycling takes place in roads with 20 mph speed limits, such roads account for just under 20% of injury points.

**Table 2:** Descriptive characteristics of injury and control points, in relation to area, road and travel volume variables.

		N (%) injury points	N (%) control points
Region	Outer	1894 (30.3%)	1621 (26.8%)
	Inner	4350 (69.7%)	4425 (73.2%)

Area income deprivation (national quintiles)	Quintile 1 (richest)	941 (15.1%)	1032 (17.1%)
	Quintile 2	792 (12.7%)	853 (14.1%)
	Quintile 3	1214 (19.4%)	1135 (18.8%)
	Quintile 4	1895 (30.4%)	1716 (28.4%)
	Quintile 5 (poorest)	1402 (22.5%)	1310 (21.7%)

Road type	Residential	734 (11.8%)	1262 (20.9%)
	Tertiary	628 (10.1%)	768 (12.7%)
	Secondary	455 (7.3%)	355 (5.9%)
	Primary	4133 (66.2%)	3134 (51.8%)
	Unclassified	294 (4.7%)	527 (8.7%)

Speed limit (mph)	20	1174 (18.8%)	1721 (28.5%)
	30	4966 (79.5%)	4208 (69.6%)
	40+	104 (1.7%)	117 (1.9%)

Bus lane	No	4886 (78.3%)	4827 (79.8%)
	Yes	1358 (21.8%)	1219 (20.2%)

Junction	No	1300 (20.8%)	2985 (49.4%)
	Yes	4944 (79.2%)	3061 (50.6%)

Motor vehicles per day on road segment	<2000	870 (13.9%)	1566 (25.9%)
	2000–9999	1701 (27.2%)	1795 (29.7%)
	10,000–19,999	2211 (35.4%)	1674 (27.7%)
	20,000–29,999	1116 (17.9%)	752 (12.4%)
	30,000+	346 (5.5%)	259 (4.3%)

Cycles per day on road segment	<1000	4259 (68.2%)	3776 (62.5%)
	1000–1999	1021 (16.4%)	1103 (18.2%)
	2000–2999	541 (8.7%)	574 (9.5%)
	3000–3999	253 (4.1%)	369 (6.1%)
	4000+	170 (2.7%)	224 (3.7%)

### 3.2 Distribution of control and injury points by London borough

**Fig. 2:**
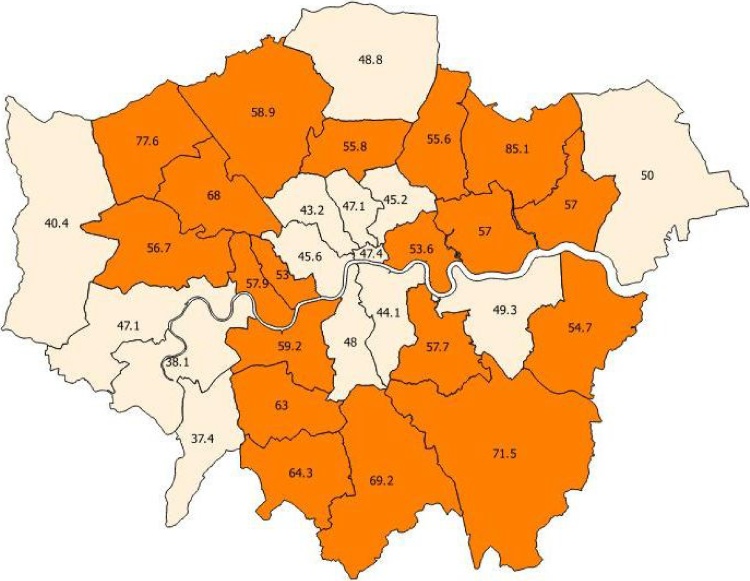
Over- and under-representation of injury points, by London borough.

Fig. 2 illustrates variation in the proportion of points in each London borough representing injuries. 50.8% of all points were injury points, and the map below displays boroughs with over- or under-representation of injury points. Inner and central boroughs tend to have lower injury risks, with some Outer boroughs (but not in Outer South-West and West London) having very high injury risks. For one borough, Redbridge, 85.1% of all points were injury points, i.e. an odds ratio of around six times higher than the safest boroughs.

### 3.3 Predictors of any cycling injury in London, adjusted models

Table 3 below presents the three models showing predictors of any cycling injury in London. Inner London appears safer in the first two models, but the point estimate is almost identical to Outer London in the maximally adjusted model. This suggests that the ‘Safety in Numbers’ effect (more cyclists on route network sections in Inner London), rather than safer road environment characteristics, is responsible for Inner London being safer than Outer London. A similar trend is apparent for deprivation, with statistically significant results in Model 2 and 3 becoming non-significant in Model 3.

**Table 3:** Area, road and travel volume predictors of any cycling injury in London: odds ratios (95% CI) (N = 12,290 points).

		Model 1: area characteristics only	Model 2: area and road segment characteristics	Model 3: area and road segment characteristics, plus cycle and motor vehicle volumes
Region	Outer	1	1*	1
	Inner	0.76 (0.57, 1.02)	0.71 (0.54, 0.94)	0.97 (0.80, 1.19)

Income deprivation	Change per 1 decile increase in deprivation	1.02 (1.01, 1.04)***	1.02 (1.00, 1.03)*	1.01 (1.00, 1.03)

Road type	Residential		1***	1***
	Tertiary		1.49 (1.28, 1.73)	1.29 (1.08, 1.54)
	Secondary		2.37 (1.98, 2.83)	1.80 (1.44, 2.24)
	Primary		2.16 (1.92, 2.44)	1.52 (1.21, 1.92)
	Unclassified		1.26 (1.05, 1.51)	1.43 (1.18, 1.72)

Speed limit(mph)	20		1***	1***
	30		1.31 (1.16, 1.48)	1.26 (1.12, 1.42)
	40+		0.89 (0.64, 1.23)	0.64 (0.46, 0.89)

Bus lane	No		1*	1
	Yes		0.88 (0.80, 0.98)	0.92 (0.83, 1.02)

Intersection	No		1***	1***
	Yes		3.50 (3.22, 3.79)	3.33 (3.07, 3.61)

Motor vehicles per day	Change per 1 logarithm increase in no. motor vehicles			1.31 (1.21, 1.42)***

Cycles per day	Change per 1 logarithm increase in number of cycles			0.82 (0.79, 0.84)***

* p<0.05.

*** p < 0.001.

In both Models 2 and 3 road type is statistically significant, with residential roads safer than other road types. The effect attenuates (apart from for ‘unclassified’ roads, likely to be diverse) in Model 3, suggesting that some of the improved safety experienced on residential roads is due to their generally lower motor traffic volumes, and generally higher cycling volumes. Junction status is very important in both models, with junctions associated with over three times the odds ratio of injury, compared to non-junction sites.

Bus lanes are associated with slightly lower injury odds (p = 0.02) in Model 2, but this effect becomes non-significant in Model 3, suggesting it may be caused by higher levels of cycling and/or lower volumes of motor traffic (controlling for Outer versus Inner London, road type and speed limit) where bus lanes exist.

In Model 3, which includes motor vehicle and cycle volume, both are important and significant to p < 0.001 – motor vehicle volume associated with increased risk, and cycle volume to reduced risk.

**Fig. 3:**
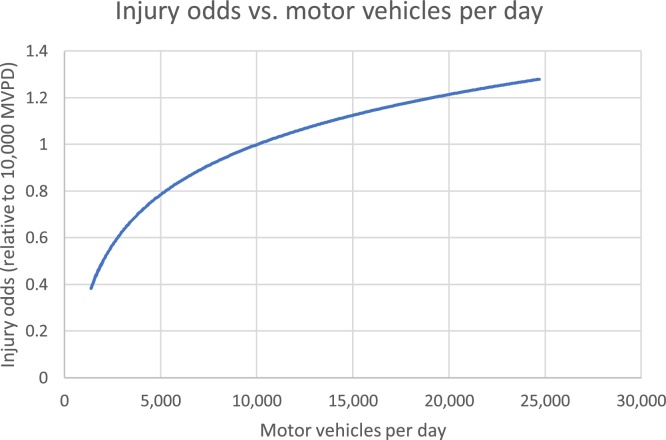
relationship of injury odds to motor vehicles per day.

Fig. 3 illustrates the relationship between injury odds and motor vehicles per day, using a scale relative to 10,000 motor vehicles per day (similar to the median level of motor traffic volume on London streets used by cyclists, as seen in Table 2 above). The graph covers the 10th to the 90th percentiles for motor vehicle volumes in the data.

**Fig. 4:**
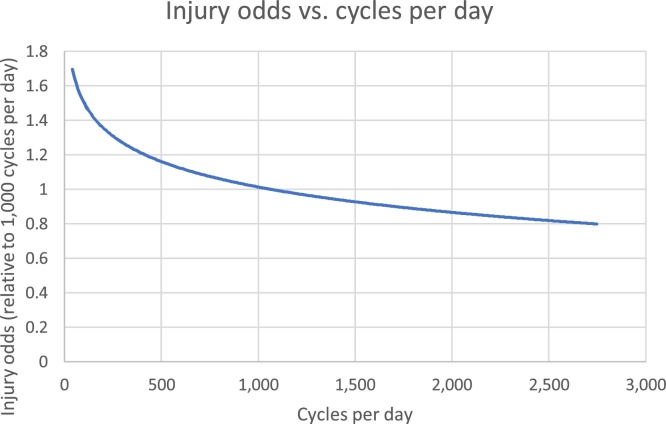
relationship of injury odds to cycles per day.

Fig. 4 shows the relationship between injury odds and cycles per day. Here the reference case is 1000 cycles per day: in the case study, this represented the 62nd percentile for control points. The graph covers the 10th to the 90th percentiles for cycle volumes in the data.

### 3.4 Junction Status

The Appendix contains tables stratified by junction status. Interactions with junction status were all non-significant (all p > 0.1), except for bus lane (p = 0.04) and cycle volume (p = 0.004). For buses, there is a weak trend towards bus lanes being slightly protective at junctions and slightly harmful away from junctions. For cycle volume, the protective effect of a higher volume of cycling is considerably stronger at non-junctions than junctions (regression coefficients 0.72 at non-junctions vs. 0.85 at junctions). This plausibly reflects measurement error at junctions when looking up cycling levels (see 2. Methods). The number of cyclists on non-intersecting links may also be relevant at junctions. Supporting this, the ‘Safety in Numbers’ effect at junctions gets stronger (and comparable to the effect at non-junctions) if using the logarithm of the mean number of cyclists across all junction links (regression coefficient 0.75 (95% CI 0.71, 0.80)).

### 3.5 KSI vs slight injuries

The injury-only model examined what predicted the injury being KSI (N = 561) vs. slight (5683). Power was low, but in the equivalent of model 3 all variables were non-significant (p > 0.05). This indicates no evidence of different predictors for KSI vs. slight injuries.

## 4 Discussion

### 4.1 Principal findings

In the maximally adjusted model, road type had a strong impact, with residential streets substantially safer than other street types. Speed limit mattered too, with 20 mph roads having 21% lower odds of injury, compared with 30mph roads. Bus lanes had a weakly protective effect due largely to higher cycling and/or lower motor traffic volumes. Motor vehicle and cycle flows mattered, with the relationship strongly statistically significant in both cases.

### 4.2 Strengths and weaknesses of the study

#### 4.2.1 Strengths

The use of a cycling model allowed analysis of injury risk in relation to exposure to different types of road environment, a methodologically strong approach still relatively rare in the literature. Unusually, road environment variables and the potential ‘safety in numbers’ effect are included within the model. Unlike previous studies (e.g. Vandenbulcke et al. (2014)) we were able to include the substantial variation (Morley and Gulliver, 2016) in motor traffic volume between the minor/residential roads cyclists often use. Further, access to TfL data allowed use of a range of infrastructure variables, some of which is not available for other parts of the UK.

#### 4.2.2 Weaknesses

One key weakness is an inability to include London’s newer high quality infrastructure, mostly built in 2015-7. Nor could analysis include all other potentially relevant factors. For instance, speed limits were used rather than actual speeds. There is a well-established relationship between actual speeds and injury risk (Elvik, 2013).

Other weaknesses are methodological. The case-control method cannot, unlike Teschke et al (2012), control for cyclist characteristics and behaviour. Hence it is possible that cyclists using apparently ‘safer’ streets are more cautious or skilled than average. Additionally, the relative importance of risk factors might change if the composition of cyclists changes, for instance, with a change in gender balance. Results are dependent on the two models used, particularly Cynemon, and uncertainties in the modelled data are not included. While random errors will tend to reduce observed differences, error resulting from systematic bias associated with explanatory variables is more problematic; for instance if Cynemon disproportionately routes cyclists onto roads of a particular type.

The Stats19 police database is widely used in injury studies to identify road traffic injuries (e.g. Grundy et al., 2009), but has limitations. Slight injuries and crashes not involving motor vehicles are particularly under-reported (Ward et al., 2006), and it is possible there is differential underreporting by our explanatory variables. Stats19 only includes injuries on or adjacent to a public highway (e.g. excluding canals). The paper, therefore, could not investigate the impact of creating routes entirely away from the public highway in London. However, matching control sites to the ITN highway network suggested such routes account for under 10% of cycle kilometres, and there is limited scope in a built-up urban environment to create new routes entirely away from the highway.

### 4.3 Meaning of the study: Possible mechanisms and implications for policy

#### 4.3.1 Safety in numbers

The paper provides new evidence for a ‘safety in numbers’ (SiN) effect at a road link/junction level, where roads with more cyclists have lower injury risk. Elvik and Bjørnskau’s recent review (2017) found a similarly consistent effect across studies, although they caution that the causes remain unclear with confounding factors usually unaccounted for. These results are consistent with, for instance, both a simple ‘physical’ explanation (more cyclists on a link means less exposure per cyclist) and a ‘behavioural’ explanation (drivers on routes with high cyclist volumes are more aware of cyclists and take more care). Unusually, the analysis separates ‘safety in numbers’ from the direct impact of infrastructural characteristics included in the model. So for example, it suggests an effect of 20 mph speed limits separate from SiN, because speed limit still has an effect after adjusting for cycling volume. By contrast, the initially similarly strong effect of cycling in Inner London disappears, as did the weaker effect associated with bus lanes.

#### 4.3.2 Road classification

In line with other studies including this variable (Teschke et al., 2012, Vandenbulcke et al., 2014) we found that road type affects injury risk. The key difference seems to be that residential roads are safer than other road types, controlling for other factors in the maximally adjusted model. Among non-residential roads, the differences are more minor and the highest risk was observed on secondary roads rather than primary roads (particularly after adjusting for traffic volume).

#### 4.3.3 Motor traffic speed limits and volumes

Separate from road classification, motor traffic volumes and speed limits seem to have independent impacts on cyclist risk. Motor traffic volumes show the expected logarithmic curve (Elvik and Bjørnskau, 2017) whereby each additional motor vehicle increases risk to other road users, but additional risk per motor vehicle falls as motor traffic volume grows. On London’s busy roads, congestion may play a role in these results, particularly as the model included speed limits but not actual speeds.

Almost all (98%) control and injury points fell on roads with 20 mph or 30mph speed limits. There was a clear reduction in injury odds in 20 mph compared to 30 mph streets. This supports the findings in Grundy et al. (2009) which concluded that the introduction of 20 mph zones in London had reduced cyclist casualties by 17% in those zones, a similar magnitude to the finding here comparing 20 mph to 30 mph streets.

However, there are counter-intuitive results in relation to roads with speed limits of 40mph or higher. In London these are often major arterial roads often with multiple lanes and barriers separating opposing streams of traffic. They represent a small proportion of the network. Cycling on these roads might be expected to be extremely dangerous.

However, in Model 2 (not adjusting for motor vehicle and cycle volumes) they appeared similar in risk to 30 mph roads, and in Model 3 (maximally adjusted) safer than 20 mph roads. Even the former seems difficult to believe. Potentially cyclists are using the footway, either legally or illegally, on some of London’s busier roads in 2013–2014 is part of the explanation. While there has generally been a consensus that footway cycling increases risk (Reynolds et al., 2009) this may not be the case for these major arterial roads with few side road junction conflicts. The model also only includes speed limits, not actually achieved speeds. Other possible factors might relate to greater skill of cyclists using 40 mph+ roads, biases in the Cynemon algorithm related to such roads, or to chance. It should also be noted that this analysis is for all injuries, even if the finding were real it would still be expected that injuries at higher speed would be more serious.

#### 4.3.4 Infrastructure

Elvik and Bjørnskau comment (2017:280) that many models do not control for infrastructure quality while where such variables have been included they ‘are at best crude indicators of infrastructure quality’. While lacking the infrastructural detail in Vandenbulcke et al. (2014), this study contributes to addressing these problems, differentiating between residential streets with low and high motor vehicle flows, identifying the speed limit on each route section, and the presence of bus lanes.

Bus lanes appeared to have a small protective effect when motor and cycle traffic volumes were not controlled for; however, the effect became insignificant in the maximally adjusted model. This suggests London’s bus lanes may offer cyclists a small reduction in risk. However, this is due to higher flows of cyclists and/or lower volumes of total motor traffic, compared to other primary roads (bus lanes being largely on such roads), rather than a protective effect from bus lanes in themselves. There is little research on cyclists in bus lanes, despite these being a major form of cycle infrastructure in the UK (Aldred et al., 2017). This finding is therefore important in highlighting the relatively limited contribution that bus lanes may make to cyclist safety, especially given their low appeal to many cyclists (De Ceunynck et al., 2015).

#### 4.3.5 Junctions

Finally, and again consistent with other studies analysing per-cyclist risk (e.g. Strauss et al., 2013; Williams, 2015), junctions are associated with substantially elevated injury odds. In general, relationships that hold across the entire dataset hold true for junctions. Thus, for instance, 20 mph speed limits remain protective.

### 4.4 Policy implications

Findings provide support for reducing speed limits from 30 mph to 20 mph, a process which continues in London and in cities and countries worldwide. They support reducing motor vehicle volumes, particularly where this can be cut to very low levels. Fig. 3 suggests for instance that reducing motor traffic volumes from 6000 to 2000 motor vehicles per day is associated with a reduction in odds of around 70%. Such a change might well increase cycling levels, as quiet streets are desirable cycling routes (Aldred, 2015). This would then further reduce risk. Fig. 4 shows that a doubling of cycling flows from 500 to 1000 per day would reduce cycling injury odds by 13%, if other factors remain constant. Injury odds ratios are however relative to cycling distance, hence benefits reduce if quieter routes involve detours.

‘Filtered permeability’ represents one way of reducing motor traffic volumes in residential areas, by removing through motor traffic. This approach is widely used in the Netherlands (Schepers et al., 2013), used in London for ‘mini-Holland’ schemes and in parts of the United States for ‘bicycle boulevards’. Such schemes sometimes raise concerns about the impact of re-routing motor traffic onto major roads. For cycling injury, the logarithmic relationship with motor traffic volumes suggests any increase in injury odds from traffic growth on already busy roads is likely to be relatively small. Further, Teschke et al.’s (2012) study suggests that also installing good quality cycle tracks on these major roads could substantially cut risks for cyclists using them.

Finally, how should we interpret the ‘safety in numbers’ effect? Woodcock et al. (2014) found cycling injury risk in Central London (where cycling is concentrated) remained much higher than in the Netherlands, so SiN does not solve safety by itself. However, the picture in low-cycling boroughs is worse, with injury odds in some Outer London boroughs to be twice as high, or greater, than in those central boroughs. Our understanding of SiN mechanisms remains limited (Elvik and Bjørnskau, 2017). However, if we know more cyclists on a route should lead to safer cyclists, this is an additional reason for policy-makers to implement interventions shown to generate new cycle trips. Recent evidence suggests cycle tracks can lead to a measurable increase in active travel uptake, for instance (Panter et al., 2016).

### 4.5 Unanswered questions and future research

Further work should combine similarly good measures of cyclist and motor vehicle volume with a detailed map of dedicated cycle infrastructure, in a context in which there is sufficient variation. Subsequent to the Cynemon 2014 estimates used, some such infrastructure now exists in London, influenced by measures used in the high-cycling, high-safety Dutch context (Schepers et al., 2017). Further exploration of the effects of shared bus lanes is warranted given the lack of other research and their widespread use in the UK.

Individual-level analysis as conducted by Teschke et al. (2012) is valuable in controlling for variations in cyclist behaviour, including the potential confounding introduced where different types of cyclist may choose different route types. This could also be used to study pedestrian risk. Finally, further analysis focusing on junctions is needed, including analysing different types of junction (e.g. roundabout versus crossroads, or major-major, minor-major, and minor-minor). This was not possible here, but would be helpful in further disentangling risks specific to junctions.

### 4.6 Conclusion

These data suggest that speed limits of 20 mph help reduce cycling injury risk, as does motor traffic reduction. The logarithmic relationship between motor traffic volumes and cycling injury risk suggests that reducing motor traffic volumes by, for example, 5000 motor vehicles a day will have much greater impact on relative injury odds on a road with 10,000 motor vehicles, than on a road with 30,000 motor vehicles. Further, building cycle routes that can generate new cycle trips will bring ‘safety in numbers’ benefits.

We would like to thank Transport for London for providing advice and support, including several datasets used here: the Cynemon model, the speed limit map, and the bus lanes map. TfL is not responsible for the analysis conducted and opinions expressed here, which remain those of the authors.

## Figures and Tables

**Table:** 

		Model 1	Model 2	Model 3
Region	Outer	1	1	1
	Inner	0.76 (0.53, 1.08)	0.72 (0.51, 1.03)	1.21 (0.96, 1.52)

Income deprivation	Change per 1 decile increase in deprivation	1.01 (0.98, 1.04)	1.00 (0.98, 1.03)	1.00 (0.98, 1.03)

Road type	Residential		1^***^	1^***^
	Tertiary		1.43 (1.11, 1.85)	1.40 (1.03, 1.90)
	Secondary		2.48 (1.86, 3.30)	2.07 (1.44, 2.99)
	Primary		2.00 (1.61, 2.47)	1.79 (1.20, 2.67)
	Unclassified		1.27 (0.94, 1.71)	1.77 (1.29, 2.41)

Speed limit(mph)	20		1^***^	1^***^
	30		1.48 (1.21, 1.81)	1.41 (1.16, 1.71)
	40+		1.18 (0.71, 1.97)	0.60 (0.35, 1.01)

Bus lane	No		1	1
	Yes		1.03 (0.85, 1.25)	1.13 (0.94, 1.37)

Motor vehicles per day	Change per 1 logarithm increase in no. motor vehicles			1.32 (1.16, 1.52)^***^

Bicycles per day	Change per 1 logarithm increase in number of bicycles			0.72 (0.68, 0.76)^***^

**Table:** 

		Model 1	Model 2	Model 3
Region	Outer	1^*^	1^*^	1
	Inner	0.74 (0.58, 0.94)	0.73 (0.57, 0.94)	0.94 (0.76, 1.15)

Income deprivation	Change per 1 decile increase in deprivation	1.03 (1.01, 1.05)^**^	1.03 (1.01, 1.05)^**^	1.02 (1.00, 1.04)^*^

Road type	Residential		1^***^	1^**^
	Tertiary		1.48 (1.23, 1.78)	1.22 (0.97, 1.53)
	Secondary		2.28 (1.81, 2.86)	1.65 (1.25, 2.19)
	Primary		2.20 (1.91, 2.54)	1.41 (1.07, 1.87)
	Unclassified		1.23 (0.97, 1.55)	1.28 (1.01, 1.61)

Speed limit(mph)	20		1^***^	1^***^
	30		1.26 (1.09, 1.46)	1.20 (1.04, 1.39)
	40+		0.77 (0.51, 1.16)	0.62 (0.41, 0.93)

Bus lane	No		1^**^	1^**^
	Yes		0.82 (0.73, 0.93)	0.84 (0.75, 0.96)

Motor vehicles per day	Change per 1 logarithm increase in no. motor vehicles			1.32 (1.20, 1.46)^***^

Bicycles per day	Change per 1 logarithm increase in number of bicycles			0.85 (0.82, 0.88)^***^

**Table:** 

		Model 1	Model 2	Model 3
Region	Outer	1	1	1
	Inner	1.02 (0.78, 1.33)	0.91 (0.69, 1.20)	1.11 (0.86, 1.44)

Income deprivation	Change per 1 decile increase in deprivation	1.01 (0.97, 1.04)	1.00 (0.97, 1.04)	1.00 (0.96, 1.03)

Road type	Residential		1^***^	1^*^
	Tertiary		1.61 (1.09, 2.37)	1.54 (0.99, 2.41)
	Secondary		2.55 (1.65, 3.93)	2.30 (1.36, 3.87)
	Primary		2.60 (1.91, 3.54)	2.31 (1.37, 3.90)
	Unclassified		1.36 (0.84, 2.18)	1.48 (0.92, 2.40)

Speed limit(mph)	20		1	1
	30		1.09 (0.84, 1.40)	1.04 (0.81, 1.32)
	40+		1.04 (0.54, 2.02)	0.81 (0.41, 1.58)

Bus lane	No		1	1
	Yes		0.85 (0.67, 1.07)	0.89 (0.70, 1.12)

Junction	No		1^***^	1^***^
	Yes		2.96 (2.42, 3.63)	2.83 (2.30, 3.47)

Motor vehicles per day	Change per 1 logarithm increase in no. motor vehicles			1.15 (0.96, 1.37)

Bicycles per day	Change per 1 logarithm increase in number of bicycles			0.86 (0.81, 0.91)^***^

## Appendix A

### Appendix A.1

Area, road and travel volume predictors of any cycling injury in London at non-junction points (N = 4285 points).

^*^p < 0.05, ^**^p < 0.01, ^***^p < 0.001.

Area, road and travel volume predictors of any cycling injury in London at junction points (N = 8005 points).

^*^p < 0.05, ^**^p < 0.01, ^***^p < 0.001.

Area, road and travel volume predictors of KSI cycling injury in London (N = 6607 points, of which 561 injury and 6046 control).

^*^p < 0.05, ^**^p < 0.01, ^***^p < 0.001.

## Footnotes

1 Teschke et al. (2012) did include cycling volumes to test for ‘safety in numbers’, surprisingly given usually positive findings, this variable had a small negative impact.

2 Cynemon models commute and non-commute purposes (e.g. shopping, business, education), but is less well able to represent purely leisure cycling.

3 While some links are one-way, Cynemon models where cyclists are likely to ride, and thus even one-way links can have a modelled two-way flow.

4 QGIS is open source geographical software allowing the manipulation and analysis of vector (point, line, polygon) and raster data. http://www.qgis.org/en/site/

5 There was relatively little change to London’s cycling infrastructure between 2013 and 2014.

6 Personal communication, TfL.

7 However, in 2013–2014 there might not have been sufficient high quality cycle infrastructure in London to detect any effect, even had better data been available.

8 Police attribution of junction status is somewhat subjective tending to be done from a sketch plan and potentially influenced by an officer’s view of whether the junction contributed to the incident: personal communication, Metropolitan Police officer involved in vulnerable road user safety programmes.

## References

[bib0005] Aldred R. (2015). Adults’ attitudes towards child cycling: a study of the impact of infrastructure. Eur. J. Transp. Infrastruct. Res..

[bib0010] Aldred R., Best L., Jones P. (2017). Cyclists in Shared Bus Lanes: Could There Be Unrecognised Impacts on Bus Journey Times?. http://www.icevirtuallibrary.com/doi/abs/10.1680/jtran.16.00072.

[bib0015] Chen P., Shen Q. (2016). Built environment effects on cyclist injury severity in automobile-involved bicycle crashes. Accid. Anal. Prev..

[bib0020] De Ceunynck T., Dorleman B., Daniels S., Laureshyn A., Brijs T., Hermans E., Wets G. (2015). Sharing is (s)caring? Interactions between buses and bicyclists on bus lanes shared with bicyclists. Proceedings of the 28th ICTCT Workshop.

[bib0025] Dozza M. (2017). Crash risk: how cycling flow can help explain crash data. Accid. Anal. Prev..

[bib0030] Elvik R. (2013). A re-parameterisation of the Power Model of the relationship between the speed of traffic and the number of accidents and accident victims. Accid. Anal. Prev..

[bib0035] Elvik R., Bjørnskau T. (2017). Safety-in-numbers: a systematic review and meta-analysis of evidence. Saf. Sci..

[bib0040] Grundy C., Steinbach R., Edwards P., Green J., Armstrong B., Wilkinson P. (2009). Effect of 20 mph traffic speed zones on road injuries in London, 1986-2006: controlled interrupted time series analysis. BMJ.

[bib0045] Jerrett M., Su J.G., MacLeod K.E., Hanning C., Houston D., Wolche J. (2016). Safe routes to play? Pedestrian and bicyclist crashes near parks in Los Angeles. Environ. Res..

[bib0050] Johnson M., Oxley J., Newstead S., Charlton J. (2014). Safety in numbers? Investigating Australian driver behaviour,knowledge and attitudes towards cyclists. Accid. Anal. Prev..

[bib0055] Kaplan S., Vavatsoulas K., Prato C.G. (2014). Aggravating and mitigating factors associated with cyclist injury severity in Denmark. J. Saf. Res..

[bib0060] Miranda-Moreno L., Strauss J., Morency P. (2011). Disaggregate exposure measures and injury frequency models of cyclist safety at signalized intersections. Transp. Res. Rec..

[bib0065] Morgan A.S., Dale H.B., Lee W.E., Edwards P.J. (2010). Deaths of cyclists in london: trends from 1992 to 2006. BMC Public Health.

[bib0070] Morley D.W., Gulliver J. (2016). Methods to improve traffic flow and noise exposure estimation on minor roads. Environ. Pollut..

[bib0075] Nordback K., Marshall W.E., Janson B.E. (2014). Bicyclist safety performance functions for a U.S. city. Accid. Anal. Prev..

[bib0080] Pai C.-W., Jou R.-C. (2014). Cyclists’ red-light running behaviours: an examination of risk-taking,opportunistic, and law-obeying behaviours. Accid. Anal. Prev..

[bib0085] Panter J., Heinen E., Mackett R., Ogilvie D. (2016). Impact of new transport infrastructure on walking, cycling, and physical activity. Am. J. Prev. Med..

[bib0090] Reynolds C.C.O., Harris M.A., Teschke K., Cripton P.A., Winters M. (2009). The impact of transportation infrastructure on bicycling injuries and crashes: a review of the literature. Environ. Health.

[bib0095] Schepers P., Heinen E., Methorst R., Wegman F. (2013). Road safety and bicycle usage impacts. Eur. J. Transp. Infrastruct. Res..

[bib0100] Schepers P., Twisk D., Fishman E., Fyhri A., Jensen A. (2017). The Dutch road to a high level of cycling safety. Saf. Sci..

[bib0105] Silla A., Ledena L., Rämäa P., Scholliers J., Van Noort M., Bell D. (2017). Can cyclist safety be improved with intelligent transport systems?. Accid. Anal. Prev..

[bib0110] Strauss J., Miranda-Moreno L.F., Morency P. (2013). Cyclist activity and injury risk analysis at signalized junctions: a Bayesian modelling approach. Accid. Anal. Prev..

[bib0115] Teschke K., Harris M.A., Reynolds C.C., Winters M., Babul S., Chipman M. (2012). Route infrastructure and the risk of injuries to bicyclists: a case-crossover study. Am. J. Public Health.

[bib0120] Transport for London (2011). Pedal Cyclist Collisions and Casualties in Greater London. http://content.tfl.gov.uk/pedal-cyclist-collisions-and-casualities-in-greater-london-sep-2011.pdf.

[bib0125] Transport for London (2017). Cynemon - Cycling Network Model for London, Presentation by Aled Davies to Cycling@Teatime, March 2017.

[bib0130] Vandenbulcke G., Thomas I., Int Panis L. (2014). Predicting cycling accident risk in Brussels: a spatial case-control approach. Accid. Anal. Prevent..

[bib0135] Vanparijs J., Meeusen R., de Geus B. (2015). Exposure measurement in bicycle safety analysis: a review of the literature. Accid. Anal. Prev..

[bib0140] Ward H., Lyons R., Thoreau R. (2006). Under-reporting of Road Casualties – Phase 1, Road Safety Research Report No. 69.

[bib0145] Whitfield G.P., Ussery E., Riordan B., Wendel A.M. (2016). Association between user-generated commuting data and population-representative active commuting surveillance data — Four Cities, 2014–2015. CDC.

[bib0150] Williams T. (2015). Investigating Characteristics in a Spatial Context That Contribute to Where Bicycle Accidents Occur.

[bib0155] Winters M., Teschke K., Grant M., Setton E., Brauer M. (2010). How far out of the way will we travel? Built environment influences on route selection for bicycle and car travel. Transp. Res. Rec. J. Transp. Res. Board.

[bib0160] Woodcock J., Tainio M., Cheshire J., O’Brien O., Goodman A. (2014). Health effects of the London bicycle sharing system: health impact modelling study. BMJ.

